# Organizational perspectives on the impacts of scaling up overdose education and naloxone distribution in Kentucky

**DOI:** 10.1186/s13722-025-00553-2

**Published:** 2025-03-14

**Authors:** Hannah K. Knudsen, Sandra Back-Haddix, Shaquita Andrews-Higgins, Michael Goetz, Olivia A. Davis, Douglas R. Oyler, Sharon L. Walsh, Patricia R. Freeman

**Affiliations:** 1 Department of Behavioral Science and Center on Drug & Alcohol Research, University of Kentucky, 845 Angliana Avenue, Room 204, Lexington, KY 40508 USA; 2 Substance Use Research Priority Area, University of Kentucky, 845 Angliana Avenue, Lexington, KY 40508 USA; 3 College of Medicine-Northern Kentucky Campus, University of Kentucky, 1 Nunn Drive, Highland Heights, Kentucky, KY 41099 USA; 4 Department of Pharmacy Practice and Science, College of Pharmacy, University of Kentucky, Lexington, KY 40536 USA; 5 Department of Pharmacy Practice and Science, Center for the Advancement of Pharmacy Practice, College of Pharmacy, University of Kentucky, Lexington, KY 40536 USA

**Keywords:** Naloxone, Overdose education and naloxone distribution, Opioid overdose, Evidence-based practice, Stigma

## Abstract

**Background:**

Efforts to scale up overdose education and naloxone distribution (OEND), an evidence-based practice for reducing opioid overdose mortality, was a major focus of the HEALing Communities Study (HCS). The aim of this analysis is to describe the qualitative perspectives of partner organizations regarding the impacts of implementing OEND in a state that used a naloxone “hub with many spokes” model for scaling up this strategy.

**Methods:**

Small group (*n* = 20) and individual (*n* = 24) qualitative interviews were conducted with staff from 44 agencies in eight Kentucky counties that implemented OEND from April 2020 to June 2022. Interviews were conducted between 6 and 8 months after the end of the intervention. Initial deductive coding used the reach, effectiveness, adoption, implementation, and maintenance (RE-AIM) framework, and then additional inductive sub-coding focused on passages within the OEND Effectiveness code. Thematic analysis was then utilized to identify themes regarding the impacts of implementing OEND.

**Results:**

Participants identified multi-level impacts of implementing OEND. At the individual-level, participants described lives being saved, greater access to naloxone for individuals served by the agency, reduced stigma toward OEND by clients, and greater client-level self-efficacy to respond to overdoses. Organizational impacts included improved staff readiness for overdose response, enhanced clinical relationships between staff and clients, and reduced staff stigma. Participants described positive impacts on their organizational networks and clients’ social networks. Community-level impacts included greater overall access and reduced stigma toward OEND.

**Conclusions:**

These qualitative data revealed that staff from agencies involved in a community-wide effort to scale up OEND perceived multi-level benefits, including saving lives, reducing stigma, improving naloxone access, and enhancing staff and client readiness, while strengthening organizational and community networks.

**Trial registration:**

ClinicalTrials.gov, NCT04111939. Registered 30 September 2019, https://clinicaltrials.gov/ct2/show/NCT04111939

**Supplementary Information:**

The online version contains supplementary material available at 10.1186/s13722-025-00553-2.

## Background

The United States (US) opioid epidemic continues to negatively impact many communities, with the most recent data indicating that 107,941 individuals died from drug overdoses with 81,806 of these from opioid overdose specifically in 2022 [1]. Recent national survey data have revealed that 42% of American adults know someone who has died from a drug overdose, and one in eight report life disruptions due to a fatal drug overdose [2]. Within the US, Kentucky has experienced rising opioid overdose rates in recent years, as indicated by an increase of 44% in suspected opioid overdose encounters by emergency medical services from 2018 to 2021 [3]. Overdose deaths in Kentucky continued to rise during the COVID-19 pandemic [4, 5], particularly in rural counties [6].

To reduce opioid overdose deaths, scaling up evidence-based practices (EBPs) has been strongly recommended by numerous stakeholders [7–9]. Overdose education and naloxone distribution (OEND) is highly effective at reversing opioid overdoses, thereby reducing mortality at the individual level [10–14]. At the community level, greater naloxone saturation in terms of population-adjusted units distributed is associated with lower rates of opioid overdose mortality [15, 16], and modeling studies indicate that scaling up OEND would be highly impactful at reducing opioid-related deaths [7, 17, 18]. However, access to naloxone has been challenging during much of the opioid epidemic due to cost, stigma, limited implementation of OEND in communities, and varying state and organizational policies, resulting in racial and ethnic inequities in naloxone access [19–24].

As part of the HEALing (Helping to End Addiction Long-term^®^) Communities Study (HCS) [25], the Kentucky research team worked with community coalitions and partner organizations to scale up OEND across eight Kentucky counties in a diverse array of organizational settings [26]. This effort involved a phased series of implementation strategies, including a centralized naloxone “hub” and a team of implementation facilitators who worked with partner organizations (“many spokes”) throughout the implementation period [27]; of note, “hub and spoke” models have been previously used to scale up access to medications for opioid use disorder (MOUD) [28]. The HCS includes the RE-AIM (reach, effectiveness, adoption, implementation, and maintenance) implementation science framework [29, 30], and previous publications have reported the outcomes of reach, adoption, and maintenance for Kentucky’s OEND effort [27, 31]. Of 209 organizations contacted about the opportunity to implement OEND through a partnership with HCS, 69% adopted the HCS OEND program, defined by receiving at least one naloxone shipment. Of these, 88% agreed to maintain OEND by transitioning to a state-funded naloxone program after the study [27]. In terms of reach, partner organizations distributed nearly 40,822 units of naloxone (with a unit constituting a two-dose package) with a mean of 281 units per partner organization. Partner organizations voluntarily collected OEND recipient demographic data, which is important for considering issues around equity of reach [32]. Although OEND recipients were more likely to be female and somewhat younger than overdose decedents in these counties, differences by race and ethnicity were not significant [31]. Nearly 70% of OEND recipients had witnessed an overdose and 41% had a personal history of experiencing an overdose.

Although the clinical efficacy of naloxone in reversing opioid overdoses is well-documented, it is less clear how partner organizations perceived the effectiveness of implementing OEND. As noted by Holtrop and colleagues [33], qualitative methods can be particularly useful in understanding the impacts of implementing an EBP, including both anticipated and unanticipated impacts. Although RE-AIM typically frames effectiveness in terms of clinical effectiveness of the EBP at the individual-level, the implementation process may have multi-level impacts on EBP recipients, individual staff, the organization, and potentially the broader community. While there is substantial qualitative literature examining perspectives and experiences with OEND among persons who use drugs [34–50], much of this research has been conducted in the absence of structured implementation efforts (i.e., as OEND has naturally diffused in communities). Other qualitative OEND studies that have focused on organizational stakeholders have typically focused on single professions, such as pharmacists [51–53], first responders [54–57], harm reduction staff [58], and medical providers [59–61] rather than multi-sectoral community-wide OEND implementation efforts. To address this gap, the aim of this study is to describe the impacts of implementing OEND as reported by partner organizations in eight Kentucky counties.

## Methods

### Study design

The HCS is a multi-site, parallel group, cluster randomized wait-list controlled trial that tests the Communities That HEAL (CTH) Intervention’s effect on opioid overdose deaths by comparing 34 communities randomized to Wave 1 (intervention) to 33 communities randomized to Wave 2 (waitlist control) in four states (Kentucky, Massachusetts, New York, and Ohio) [25]. Kentucky’s communities were counties, and four of the eight Wave 1 counties were rural; other county-level sociodemographic characteristics are presented in Supplementary Material 1. Wave 1 communities received the CTH intervention from January 1, 2020, through June 30, 2022. In brief, the CTH intervention emphasizes community engagement via coalitions that work through a multi-phase process to prioritize EBPs for implementation at various community organizations using the Opioid-overdose Reduction Continuum of Care Approach (ORCCA) [26, 62–64]. OEND is one of three categories within the ORCCA. Through HCS-supported technical assistance and resources, coalitions and partner organizations worked to implement OEND across a wide range of settings. In addition, coalitions and the research teams worked to deploy communication campaigns to support EBP scale-up, including OEND [65, 66]. Although the CTH did not significantly reduce opioid overdose deaths in the intervention communities relative to the waitlist control communities [67], the CTH intervention was significantly associated with an increase in community-level rates of naloxone distribution [68]. The study protocol (Pro00038088), including the design of the interviews for this analysis, was approved by Advarra Inc., the HCS’s single Institutional Review Board, and the study was registered at ClinicalTrials.gov (NCT04111939).

The Kentucky team developed a centralized naloxone “hub with many spokes” approach to implementing OEND in the eight Wave 1 Kentucky communities [27, 31]. The Naloxone Hub, which was tasked with naloxone ordering, labelling, shipping no-cost naloxone to partners, and expiration date monitoring, was located at the University of Kentucky. The “many spokes” were partner organizations located in the eight Wave 1 communities. Multi-faceted implementation strategies were utilized to support OEND implementation, with implementation facilitation representing a major strategy to engage and work with organizations throughout the Wave 1 period. The first partner organization adopted OEND in April 2020, and implementation continued through June 2022. During a six-month sustainment phase (July 2022 to December 2022), the Naloxone Hub continued to provide final shipments to partner organizations, with Implementation Facilitators continuing to assist agencies in transitioning to the state’s naloxone program.

### Data collection

To gather information about how internal, external, and intervention-related factors may have facilitated or impeded implementation and sustainment of these EBPs, qualitative semi-structured interviews were conducted with a purposive sample of Wave 1 partner organizations. Data collection occurred from January 2023 to March 2023, approximately 6–8 months after the Wave 1 CTH intervention period ended. It was anticipated that approximately 2–3 staff from a given organization would participate in the interview, with some interviews conducted with a single individual depending on interest and availability within the organization. To identify potential interviewees, internal databases were used to identify a purposive sample that included a range of organizations that were (1) in rural and urban communities, (2) represented a mix of organizations with and without affiliated members serving on the eight community coalitions, and (3) inclusive of the three primary sectors—health care, behavioral health, and criminal legal system—prioritized within HCS. This purposive sampling strategy, rather than a census-based approach, was developed by research teams from the four states due to resource constraints. Given the complexities of qualitative data collection and analysis in a large multi-state study as described in McAlearney et al. [69], a saturation-based approach to sampling was deemed unfeasible. To better understand the implementation of EBPs during HCS and sustainment, an interview guide, informed by the RE-AIM framework, was developed by implementation scientists affiliated with the four research teams through a series of cross-site meetings. All four research teams conducted interviews, but this analysis focuses only on Kentucky’s data because of its unique “hub with many spokes” model for implementation.

Interviews were conducted by Kentucky’s Implementation Facilitators, most of whom were involved in working with organizational partners to implement OEND during the Wave 1 intervention. Interviewers contacted potential participants (*n* = 123) by email or telephone to explain the purpose of this data collection and ask if they were willing to participate in a small group or individual interview to be conducted by video conference or by telephone. After providing verbal informed consent, participants were asked open-ended questions about their experiences with implementing OEND during the CTH intervention and post-intervention as well as the impacts of their partnership with HCS. Example questions included: *“How would you describe the overall impact of providing OEND during HCS on your organization?”* and *“How would you describe the overall impact of providing OEND during HCS on [your patients/your clients/the people that it serves]?”* Because of the semi-structured nature of the interview, interviewers probed for clarity or greater detail. Individuals who participated were eligible to receive $50 in the form of Amazon gift card, unless state, government, and employer regulations or policies did not permit employees to receive compensation for participating in research. Small group and individual qualitative semi-structured interviews were conducted with 70 individuals employed by 44 OEND partner organizations. Of the 53 individuals who did not participate, 14 individuals refused, and the remainder did not respond to repeated invitations. All interviews were audio-recorded and professionally transcribed.

### Data analysis

As part of a four-state consensus-based deductive coding process [69, 70], transcripts were initially coded in *NVivo 12* using a codebook that included codes based on the RE-AIM model for OEND [29, 30]. In brief, two individuals from each of the four research teams (*n* = 8) were involved in coding the same transcripts until consensus was reached. Then, within each team, coders were trained and initially coded the same transcripts until consensus was reached; for the Kentucky team, consensus was reached after three transcripts, and the remaining transcripts were coded independently.

To address our aim of describing the impacts of scaling up OEND, passages within the OEND Effectiveness code were extracted for additional inductive sub-coding. The lead author (HK) reviewed all passages (*n* = 119) before drafting an initial codebook of sub-codes that contained new code definitions and inclusion/exclusion criteria. A small group of coders (HK, SAH, SBH, MG, OD) applied the initial draft of the new codes to a subset of passages to ensure consensus in application of the codes, to identify revisions needed to increase clarity of the codebook, and to determine whether additional themes emerged from the data which would warrant additional new codes. The codebook for this analysis is presented in Supplementary Material 2, and our consolidated criteria for reporting qualitative research (COREQ) checklist [71] is included as Supplementary Material 3.

Once the inductive coding process was complete, the coding team conducted a thematic analysis [72, 73] to identify themes regarding the impacts of implementing OEND. To enhance rigor, the coding team reviewed code reports independently to identify themes, and then during meetings, the team used a consensus-based process to discuss the themes and identify representative passages of each theme [74]. The inductive coding process revealed the multi-level nature of the impacts of scaling up OEND, similar to socio-ecological models [75], which informed how we organized our results.

## Results

In our thematic analysis, we found that OEND partner organizations described impacts of implementation that spanned multiple levels. Many interviewees described direct impacts of OEND on the people served by their agency, including lives directly saved by naloxone and improved access to naloxone. Impacts of OEND implementation also occurred at the organizational level in terms of staff perceptions about self-efficacy, the quality of clinical relationships with patients/clients, and staff stigma. Finally, OEND implementation by partner organizations also had cascading effects on relationships within their organizational networks and the community at large. Representative passages of these themes are presented, with additional passages included in Supplementary Material 4. Characteristics of partner organizations and interviewees are presented in Table 1.

**Table 1:** Organization (*n* = 44) and interviewee (*n* = 70) characteristics

	% (*N*)
Organizations’ geography	
Rural community	43.2% (19)
Urban community	56.8% (25)
Organizational sector	
Behavioral health (e.g., medications for opioid use disorder, counseling-based substance use disorder treatment) and community services (e.g., domestic violence programs, shelters for people who are unhoused)	59.1% (26)
Criminal legal system (e.g., jails, specialty court)	6.8% (3)
Healthcare (e.g., syringe service programs embedded in health departments, primary care, emergency medical services)	34.1% (15)
Interviewees’ geography	
Rural community	40.0% (28)
Urban community	60.0% (42)
Interviewees’ organizational sector	
Behavioral health and community services	64.3% (45)
Criminal legal system	4.3% (3)
Healthcare	31.4% (22)
Age	
18–34 years	24.3% (17)
35–49 years	42.9% (30)
50–64 years	28.6% (20)
65–74 years	4.3% (3)
Sex	
Female	84.3% (59)
Male	15.7% (11)
Ethnicity	
Hispanic/Latino/a/e	7.1% (5)
Non-Hispanic	92.9% (65)
Race	
White	90.0% (63)
Black	7.1% (5)
Asian	1.4% (1)
Missing	1.4% (1)
Educational attainment	
High school degree or equivalent	1.4% (1)
Some college, no degree	8.6% (6)
Associate degree	8.6% (6)
Bachelor’s degree	28.6% (20)
Master’s degree	48.6% (34)
Doctoral degree	1.4% (1)
Professional degree (e.g., MD, DDS)	2.9% (2)

*Note: Percentages may not sum to 100% due to rounding*

### Individual-level impacts of OEND implementation

*Lives Saved and Increased Access.* When participants involved in implementation were asked about the impacts of OEND, many participants shared that it was a key factor in life-saving efforts– not only with their patients/clients but also other individuals in the community. Partner organizations who implemented OEND heard many stories about how someone had received OEND at their organization and subsequently, they were able to reverse an overdose in their community with HCS-provided naloxone which saved a person’s life. One participant shared how an OEND recipient did not know what would have happened if they had not received naloxone a few weeks prior and had been prepared to respond to an overdose:

“We actually had a guy, a client that had to use some [naloxone] that we gave to him on a neighbor a few weeks ago, and he’s like, ‘If y’all hadn’t gave me that, I don’t know what would’ve happened.’ So yeah, we still hand it out left and right (01082401).”

Another participant noted how a client used HCS-provided naloxone to save the life of his girlfriend:

“Oh, it’s actually been really good. I’ve enjoyed doing it, but I’ve got a lot of good responses. We’ve actually had participants come back and say they didn’t use theirs, but they gave it to somebody that it was used. And actually, we did have one participant, his girlfriend did overdose, and he had it, and he hit her with both shots of Narcan and brought her back before the paramedics got there (01082056).”

Partner organizations shared that, through resources provided by HCS, their staff were able to expand access to OEND. In part, enhanced access to naloxone was facilitated by staff at OEND partner organizations being able to build positive, trusting connections with their patients/clients which created an environment where patients/clients felt comfortable enough to relay their need for replacement units of naloxone. One participant shared how their team provided a safe space so that patients/clients knew they could request additional naloxone as needed:

“So just knowing that they have that safe, reliable place with no stigma, no judgement that they can replenish their supply, or they can get some for a friend or whatever. I think that has made a huge impact. And just seeing the success in the provider teams and hearing the patients’ successes and knowing that they feel comfortable coming and asking for it, I feel sure that we’ll continue it (1070983).”

*Patient/Client Stigma and Perceptions of Safety/Self-Efficacy*. Participants also readily discussed program impacts on stigma from the patient/client perspective and the evolving attitudes regarding OEND by those the partner organizations served. Positive interactions with organization staff, however, were reported to help destigmatize overdose educational efforts and carrying naloxone. Requesting HCS-provided naloxone became more acceptable in the eyes of many patients as they continually built trust with the partner organization and staff:

“Now we do our annual assessments and things like that, we ask, “Do you or someone around you have access to Narcan?” That is something we’re trying to stay proactive on it. I think it just kind of didn’t really put this negative stigma of, ‘Oh my goodness, if I have this, that just looks bad on me. Everybody thinks it’s for me.’ So, I think having it here definitely helped, just from what I’ve seen from a clinical patient standpoint (01094135).”

Patient attitudes towards OEND were also driven by increased feelings of empowerment and self-efficacy in the event that they witnessed an overdose. Some participants believed patient attitude changes were partially attributed to this more active role in their own recovery and in protecting their community:

“Interviewee 1: Lives have been saved. The women that we serve feel a lot safer and prepared to help not only themselves, but others who are around them. I think they’re taking a more active role in making sure their friends and acquaintances are safe.”

Interviewee 2: On a personal level for them. It also is giving them some sense of responsibility almost knowing that they have that, they know how to use it, and they could help somebody else (01074358).”

In addition to the impacts of destigmatization, patient perspectives were heavily influenced by feelings of increased safety brought on by receiving OEND. Prior interactions with overdose events, whether experienced or witnessed, affected perceptions of personal security, regardless of where a patient was in their recovery. Carrying naloxone was viewed as a substantial safety measure by patients should they encounter or experience another overdose event:

“And the stories that we would hear of individuals that had been in maintenance but still came and would get Narcan or replace their Narcan or whatever, just to say, ‘I know it saved my life, so I always want to have it handy, to be able to save somebody else’s (01152446).”

*Social Networks of Patients/Clients.* OEND was found to have ripple effects on individuals beyond the partner organizations’ immediate patient population. This was a result of the dissemination of overdose intervention knowledge and naloxone supplies via patients to their social connections in the community. One participant commented on the ease by which an established patient could secure naloxone for another person without directly involving a naloxone provider or forfeiting the other individual’s anonymity:

“But the fact that they could also get Narcan for a loved one without telling a provider or sharing that information with anyone; knowing that they could come to use and get it anonymously; they could encourage their family members and friends to do it (01151015).”

As impactful as HCS-provided naloxone could be alone, other participants were quick to point out that overdose response knowledge and education provided much-needed awareness in a region heavily impacted by the opioid epidemic. Life-saving measures were made available on a larger scale simply through word-of-mouth efforts:

“So, when we provided them with the words to be able to tell people what Narcan was, they defined it to other people, and so they educated people as well in their community. I know for sure we had at least one that said, ‘Hey, it’s not for me, it’s for somebody else.’ I said,‘I don’t care who it’s for. Just tell her you need another one (01083214).’”

### Organizational impacts of implementing OEND

*Staff Perceptions of Safety/Self- Efficacy.* Participants discussed not only opioid overdose deaths occurring in the community, but their experiences with the deaths of people served by their agency before implementing OEND. Even though overdose deaths were still occurring in the community, participants shared that implementing OEND improved their agency’s efforts to help prevent the event of fatal opioid overdoses, which led to staff feeling more confident in their prevention efforts:

“We’ve experienced a lot of death in the five years that I’ve been here. We’ve experienced a lot of death, and I have never felt like those deaths were on me. Does that make sense? I’ve always been able to understand in whatever, but now more so than ever, I feel like we are absolutely doing everything that we can to prevent somebody’s life from ending that doesn’t need to end. It [OEND] gives me that much confidence (01083214).”

Being equipped and knowledgeable about how to respond to an opioid overdose increased the comfortability level and sense of security of agency staff:

“And I’ll tell you, it [OEND] gives me a sense of security because there are times that people will use and come in these doors. So that it gives you a sense of security knowing that you’re equipped to handle [it]. You would think that this might not be the place that someone may overdose or maybe be highly intoxicated, but unfortunately there’s times that that happens. Just knowing that we’re a little more equipped or prepared if that happens is really big (01022768).”

*Clinical Relationships.* As the staff’s comfort increased because of providing OEND, participants described how impactful and therapeutic OEND delivery could be for clinical relationships with their patients. Participants discussed ways the relationships were built through the delivery of OEND, which let their patients know that agency staff were there for them in a safe space throughout their recovery journey:

“From the organization standpoint, I would say that it’s [implementing OEND] impacted us favorably because it helps our patients know that we really are here to help them, and we want them to get better, even through different stages of recovery that they may be in, we’re still going to be here (01022776).”

In addition to staff building the therapeutic relationship, participants discussed providing tools to assist patients throughout their recovery journey by adding OEND to their individual and group therapy, peer support, and case management services:

“I think that was the biggest impact [of OEND] is really building just that relationship with them to let them know we’re not judging you. We want everybody to be safe and feel like they have the tools that they need for wherever they are on their recovery journey. And this is just one of those tools. Same as therapy or group or whatever it may be, peer support, whatever it may be, case management. I guess it’s just another tool that we can offer you in that journey (01152446).”

One participant described the clinical relationship as being positively impacted by OEND, even in the relatively brief interactions of an ambulance ride; such conversations, in turn, created a way for the agency to be able to serve their patients in a greater capacity to offer additional recovery resources:

“But I think you can start to see some of the seeds being planted amongst the personnel as they’re having conversations with individuals in the back of the ambulance, ‘Hey we gave you Narcan, you’re going to have a team that’s coming out to see you. Listen to what they have to say because they’re trying to help you.’ And that’s definitely a big shift that allows us to serve people better and opens the door for people in this space to be served because they’re more willing to have a conversation (01072642).”

*Staff Stigma Related to OEND.* Another way that staff were positively impacted by OEND implementation at their agencies was through stigma reduction. Participants discussed the stigma surrounding OEND being present within local community organizations. However, through the implementation of OEND, the culture at the agency shifted, along with making progressive changes with their processes and procedures. One participant discussed the staff perceptions of harm reduction had changed to a more positive outlook:

“And I think the conversations that they have and the conversations they drive has really changed or is changing the culture as well within the fire department. Last year, [a local detective] was part of their in-service and one of the conversations she always talked about was harm reduction–why we carry Narcan, why we hand out Narcan, why Narcan is so valuable (01072642).”

Another participant described that due to the implementation of OEND, agency staff wanted to move away from a more disciplining treatment approach to a more healing approach:

“Like I mentioned, being able to train the staff [about OEND] and start there and have that conversation with them really worked in line with the changes we wanted to make in our own staff of not being punitive and really being therapeutic. And so, it was just a great tool to be able to use with them (01152446).”

### Impacts on organizational networks

Participants discussed not only how training the staff at their own treatment agencies was valuable, but that staff felt comfortable enough to share their knowledge about the importance of OEND with others in their organizational networks. Staff being able to share their success of OEND implementation helped change the attitudes of staff in other organizations, expanding the knowledge of OEND throughout the community:

“I definitely have seen for this new year, we’ve having a lot of people wanting to do Narcan trainings. The [city department], I’m going to Narcan train all 12 supervisors that work in the [department] in [city]. I’m going to work with some business owners that are downtown that want to all group together and do Narcan. I just did [local hotel’s name] Narcan training with their staff in [city]. I have another one at a behavioral health facility tomorrow. And then I have one of the treatment houses asking me for Narcan. So that’s six people on the list. And that’s probably going to be a hundred trainings or something (01091191).”

One participant discussed because of providing OEND at their site, the information was expanded to a broader range of people outside of their local community:

“We have centers in all over the state, and so we got to the point where our staff and people at the other centers and stuff, they would reach out to me because they knew that we had the Narcan, or they would ask me where they could get some or how they could be trained. So, I think that it really impacted us as a whole company. I think that we were able to reach a lot more people other than just [county], even if it was just me giving them some education or whatnot (01152791).”

Another participant shared that because of the implementation of OEND, neighboring agency staff felt more secure knowing that a nearby agency had naloxone units available to administer on-site if needed to reverse an overdose:

“And honestly too, like [government agency] is attached to our building. They know that we have Narcan and how many times they came next door and said, ‘Hey, it looks like somebody’s passed out in the parking lot.’ I don’t know that we’ve ever had to use it, but they felt confident enough to come next door and know that we had it (01152791).”

For many agencies, implementing OEND placed them in a leadership role, where their staff influenced other organizations both locally and beyond their own community.

### Community-Level impacts of implementing OEND

*Community Access.* HCS partner organizations worked to make naloxone more widely available to everyone in the community, beyond individuals who represented the agency’s clients or patients. Previously, naloxone availability existed primarily through community events, and HCS increased the number of venues from which the community could access the overdose reversal medication. Increased accessibility removed a barrier for more community members to carry and potentially administer naloxone:

“Well, I just think that it made the difference because it eased the access. Before this began, people didn’t [know] where they were going to get Narcan. It might be at special events, but it wasn’t just always around the way that it is now. Now, I feel like people have, I mean people can get it in so many different spots across the county, and that’s going to keep people safe and alive (01032790).”

Further, while greatly increasing OEND access, HCS partner organizations made additional distribution efforts to under-served communities. According to interviewees, these purposeful efforts were successful in reaching people affected by overdoses. People shared their stories and readily accepted naloxone to prevent accidental deaths in the future:

“We didn’t set-up at [big box store], we didn’t set-up at [grocery store], we set up at the shady motels. We set up at the hood gas stations, we set up in the hood and in hollers, and it was impactful. Oh my gosh, so impactful. Getting to distribute naloxone was impactful, but hearing how many people were affected by overdoses. ‘Yes, I’d love one. My niece overdosed.’‘Yes, I’d loved one. I lost my husband a month ago.’ You know? Over and over and over. So, that was impactful (01022769).”

Targeting OEND to high-risk areas was a strategy of many partner organizations. Some agencies used data-driven decision-making to ensure they avoided redundancy and strategically allocated resources to maximize impact. Using these inputs, they could offer education and naloxone to those who needed it most:

“The social media and geofencing has been running year long, but we’re targeting specific chunks, trying not to overlap as much as we can with these other media promotions so that we can hopefully in turn go back over the year’s data and see which months did we have more of an increase in our mailed naloxone to see which promotion got the most response, like where could we get that. We would like to in the long run also use that as part of our overdose surveillance response and so that we can target communities when we see a spike in overdoses–can we quickly shift our funds and money towards promotion that we know drives the most outcome to a community (01131207).”

*Reductions in Community Stigma.* With this push to include the whole community in OEND efforts, HCS and its partners witnessed a decline in stigma about carrying naloxone and the need for education on how to reverse an opioid overdose. There was initially resistance to carrying naloxone by some in the community, but there was a positive change over time as it became more accepted by the community:

“Yeah, like I was saying, one, it changed the perspective. I know that early on there had been a lot of pushback. Not a lot, but there had been some pushback at events where they were giving out Narcan or naloxone, and then just over time, it’s just became a thing. It’s just, all of a sudden there’s not that stigma attached to it. It just is what it is (01032790).”

Recipients of OEND began to understand they could play an active role in harm reduction. Carrying naloxone became less about generalized stigma and more about helping others and a growing comfort with the realities of opioid use disorder (OUD):

“Well, you used to ask somebody if they wanted Narcan, it was like, ‘no.’ Then, you’d ask them and they’d be like, even if they didn’t need it, they knew somebody that did, and they want to help. So, that totally changed as time went on (01022769).”

Community members took the initiative to acquire naloxone from partner organizations. Even those traditionally hesitant to associate with drug use or its stigma recognized that carrying naloxone was part of community safety:

“I had an elderly gentleman call my office. He said, ‘I read this article that came in this magazine to my house.’ He said,‘I’m a pastor at a church. After reading this article,’ he goes, ‘I feel like this is something I should also be carrying being a concerned community member.’ He said, ‘I found the number–phone number–on here, and I called.’ He goes, ‘Can I also get this?’ We said,‘Absolutely’ (01131207).”

The overdose education materials provided by HCS, coupled with the communication campaigns, relayed that anyone could be a bystander witnessing an overdose and effectively respond. Traditionally, OEND programs had been focused on adults with opioid use disorder, but through increased distribution efforts and HCS education programs, the recipients of OEND diversified, and the stigma subsided to some degree:

“When you see the little old church lady who has the Narcan in her bag, who willingly takes it from, and she has the Narcan in her bag because it’s not stigmatized any longer, that’s a huge thing for me. When you have an adult child who lives with an elderly parent and the elderly parent knows how to use the Narcan just in case, that’s huge. And we had that reported multiple times (01083214).”

Through education shared around overdose prevention, the pushback and stigma that communities once saw as a major barrier began to break down, and more community members started to accept naloxone as a lifesaving, life-changing tool.

## Discussion

The HCS sought to scale up OEND in multiple organizations within communities, and qualitative interviews with partner organizations in Kentucky revealed that implementing OEND had positive impacts on multiple levels. Participants described ways that OEND positively impacted the individuals served by their agencies, and that OEND implementation had meaningful impacts on the social networks of individuals, the attitudes of staff working at the agency, inter-organizational relationships, and stigma in the broader community.

Our themes about the impacts on individual clients and patients align with findings from prior qualitative studies about OEND conducted with people who use drugs (PWUD). Similar to our findings, other studies have documented that opioid overdoses were reversed and lives were saved through the utilization of naloxone that was provided to PWUD [40]. In addition, prior qualitative research indicates that individuals who have access to naloxone are highly likely to reverse an overdose in a crisis situation [48]. By having access to this life-saving medication, previous studies point out that response behaviors to suspected opioid overdoses have significantly improved with OEND due to patients and clients feeling more prepared to respond [48]. Similar to our study findings, another preceding study pointed out that through harm reduction conversations, patients and clients are more willing to carry naloxone particularly when OEND is focused on community-level harm reduction [39]. Previous studies have also found there may be reductions in overdose deaths among individuals in clients’ social networks because of the connectedness to people in their everyday lives who continue to use drugs [48]. Having access to naloxone provided a sense of responsibility and preparedness for patients/clients because they were able to make sure their loved ones or friends were safe in the event of an overdose [37], which is similar to the responses of participants in our study.

Implementing OEND also impacted the internal dynamics within these organizations, with participants noting how implementation affected staff attitudes, increased staff members’ preparedness to respond to an overdose, and enhanced clinical relationships between staff and patients/clients. We found that staff who held positive attitudes toward OEND led to a positive outlook on how they provided overdose response training and overdose education to patients/clients. This finding aligns with prior research where staff shared positive attitudes in providing naloxone counseling as a preventive measure and emphasized the importance of naloxone counseling to raise awareness and prevent opioid-related overdose events amongst their patients/clients [45]. Additionally, OEND provided the opportunity for positive clinical relationships, with staff treating clients with professionalism, respect, and kindness, which led to an environment where clients felt welcome, accepted, and at ease with staff members. Other research has shown that many clients receiving OEND described positive experiences when staff were professional and treated patients/clients with respect which led to patients/clients feeling welcomed, accepted, and comfortable [42].

These positive impacts of implementing OEND on staff attitudes are particularly notable, as it has been repeatedly noted that stigma within the healthcare system, as embodied in negative perceptions of PWUD by healthcare team members, creates additional barriers in the recovery process [76–78]. Delays in treatment-seeking efforts, less comprehensive healthcare visits, decreased patient empowerment, and increased loss to follow-up have all been observed in conjunction with stigmatic views of healthcare professionals [79]. OEND may be a potential tool in eroding stigma towards PWUD in the healthcare system.

Community stigma about OEND has been a salient, pervasive theme in prior qualitative studies with PWUD, and many interviewees discussed stigma in the community. Previous qualitative studies have identified stigma in the community as a significant barrier to OEND implementation for a variety of reasons. In those prior studies, community members often perceived naloxone distribution as endorsing drug use rather than addressing the root cause of addiction, leading to resistance despite its life-saving potential [37, 60]. Additionally, prior research has reported the stigma associated with substance use disorders made both community members and potential recipients uncomfortable with naloxone, viewing it as merely a “band-aid” solution to the larger problem of individuals developing and needing treatment for OUD or as enabling opioid use [60]. In these studies, such stigma complicated education efforts, resulting in misunderstandings or negative reactions from individuals who may feel accused of addiction struggles, further limiting naloxone’s acceptance and use on a larger scale [34, 44, 45]. However, in our interviews which occurred after efforts to scale up OEND in multiple community locations, agency staff indicated some reductions in community stigma, particularly around greater willingness by community members to carry naloxone. It should be noted, however, that concern over how one would be perceived by the community for carrying naloxone remained in many anecdotes. This is a testament to the need for sustained efforts in tackling the deeply entwined, stigmatic beliefs that surround the support and care of PWUD. These multi-sectoral efforts to scale up OEND, coupled with communication campaigns about naloxone that were also deployed as part of the study [65, 66], may have worked synergistically to normalize carrying naloxone, thus reducing stigma to some degree.

These findings about community stigma differ from survey data collected during HCS from individuals who served on the coalitions, which reported that coalition members in intervention communities did not report a statistically significant decrease in perceived community-level stigma toward naloxone [80]. The current study interviewed partner organizations who may have had different perspectives based on implementing OEND compared to coalition members, many of whom did not work in agencies that implemented OEND. For partner organizations, the impacts may have been more observable, particularly when they engaged in community distribution efforts. In addition, these interviews were conducted about 6 months after the surveys of coalition members; it may be that more time was needed for the impacts of OEND scale-up on community stigma to be observed. Nonetheless, our study findings pointed to the impacts of OEND through social network dynamics; however, more time may still be needed for information and resources to saturate the community.

Several limitations should be noted. First, these interviews were only conducted with staff from partner organizations, so it is unknown whether similar themes would have been identified through interviews with recipients of OEND, with PWUD, particular demographic subgroups, or with a general sample of community members. Second, interviews were conducted about six months after the end of CTH intervention, so these reflections on OEND implementation represent relatively short-term impacts; it is unknown whether these impacts were sustained in the longer term, although 88% of partner organizations intended to sustain OEND by transitioning to a state-funded source of no-cost naloxone [27]. Third, it is possible that agencies with less positive experiences in implementing OEND may have been less likely to participate in these interviews, and we did not involve partner organizations in validating the themes that we identified. Finally, in some instances, interviewers and interviewees had worked together during the implementation phase of the study, which may have introduced a positive bias into interviewee responses about their experiences with implementing OEND.

## Conclusions

This study suggests implementing OEND may have significant implications at the individual, organizational, and community levels. For individuals, access to OEND saved lives. People equipped with naloxone and overdose response knowledge were empowered to intervene and to participate in harm reduction efforts. However, additional multifaceted approaches, including structural and health policy changes, are needed to further reduce the harms of the opioid epidemic [81]. HCS was multifaceted in that it encouraged communities to also scale up medications for opioid use disorder by expanding capacity, improving linkage to MOUD, and enhancing retention in care. Although many strategies were selected for implementation [26], community-level differences on the rate of practitioners with a DATA 2000 waiver who actively prescribed buprenorphine [82] or rate of Medicaid enrollees with OUD receiving behavioral therapies [83] between CTH intervention communities and waitlist control communities were not observed, pointing to how difficult it is to overcome structural barriers, particularly in healthcare systems, to achieve community-level change. In contrast to the challenges around expanding MOUD, social networks of individuals and organizations participating in HCS’s effort to scale up OEND increased the number of individuals carrying naloxone and holding the knowledge to reverse opioid overdoses. There was also evidence of a multiplying effect when individuals and organizations shared knowledge about implementing OEND in social networks, increasing the harm reduction capacity of communities.

Further research on the long-term impact of OEND implementation on individuals, organizations and communities is needed to determine whether the positive impacts found in this study are sustained. Also, as research has shown that sub-groups may respond differently to naloxone interventions [36, 50], additional research is needed on scaling up OEND to address the needs of diverse communities. Finally, future studies should examine whether sharing information about the multi-level positive impacts found in the current study may help to persuade organizations to adopt OEND.

## Electronic supplementary material

Below is the link to the electronic supplementary material.

Supplementary Material 1

Supplementary Material 2

Supplementary Material 3

Supplementary Material 4

## Data Availability

Data reported in the current study are not publicly available to protect the privacy of organizational partners.

## Abbreviations

CTH: Communities That HEAL
EBP: Evidence-based practice
HCS: HEALing (Helping to End Addiction Long-term^®^) Communities Study
KY: Kentucky
MOUD: Medications for opioid use disorder
OEND: Overdose education and naloxone distribution
ORCCA: Opioid-overdose reduction continuum of care approach
OUD: Opioid use disorder
PWUD: People who use drugs
