# Supplementary material for: Organizational perspectives on the impacts of scaling up overdose education and naloxone distribution in Kentucky
Source: Addict Sci Clin Pract. 2025 Mar 14;20:27. doi: 10.1186/s13722-025-00553-2 (PMC11907800; doi:10.1186/s13722-025-00553-2)
Supplement: Supplementary file 1 — Supplementary Material 1 [file 13722_2025_553_MOESM1_ESM.docx]

**Organizational Perspectives on the Impacts of Scaling Up Overdose Education and Naloxone Distribution in Kentucky**

**Additional File 1**

Hannah K. Knudsen, Sandra Back-Haddix, Shaquita Andrews-Higgins, Michael Goetz, Olivia A. Davis, Douglas R. Oyler,

Sharon L. Walsh, and Patricia R. Freeman

University of Kentucky

**Comparison of Wave 1 HCS-KY Counties to State Characteristics**

|  | Kentucky | Boyd | Boyle | Clark | Fayette | Floyd | Franklin | Kenton | Madison |
| --- | --- | --- | --- | --- | --- | --- | --- | --- | --- |
| Population^1^ | 4,505,836 | 48,261 | 30,614 | 36,972 | 322,570 | 35,942 | 51,541 | 169,064 | 92,701 |
| Rural/Urban^2^ | N/A | Urban | Rural | Urban | Urban | Rural | Rural | Urban | Rural |
| Median age^1^ | 39.2 | 42.1 | 40.0 | 40.9 | 35.4 | 42.9 | 40.6 | 37.9 | 34.5 |
| Race^1^ |  |  |  |  |  |  |  |  |  |
| Asian | 1.7% | 0.6% | 1.2% | 0.6% | 4.2% | 0.3% | 2.0% | 1.2% | 1.1% |
| Black | 8.0% | 2.7% | 7.2% | 4.5% | 14.9% | 0.3% | 8.7% | 4.8% | 4.3% |
| White | 82.4% | 91.8% | 82.9% | 88.1% | 68.3% | 96.9% | 81.0% | 85.2% | 86.9% |
| Other^3^ | 2.5% | 0.9% | 2.2% | 2.2% | 5.6% | 0.3% | 2.3% | 2.6% | 1.7% |
| Multiracial | 5.4% | 4.1% | 6.6% | 4.7% | 7.1% | 2.2% | 6.0% | 6.2% | 6.1% |
| Hispanic or Latino/a/e^1^ | 4.6% | 1.5% | 4.9% | 4.0% | 9.2% | 0.9% | 4.0% | 4.6% | 3.3% |
| Median household income^1^ | $61,118 | $58,327 | $56,568 | $61,878 | $66,392 | $38,723 | $62,929 | $79,392 | $65,502 |
| Bachelor’s degree or higher of adults aged 25 and older^1^ | 27.8% | 20.8% | 26.8% | 20.0% | 49.8% | 13.1% | 31.6% | 38.9% | 36.8% |
| Poverty rate^1^ | 16.4% | 17.4% | 15.0% | 15.1% | 16.0% | 29.3% | 13.3% | 10.3% | 13.0% |
| Employment rate^1^ | 57.4% | 48.9% | 50.5% | 56.8% | 65.5% | 39.2% | 58.9% | 65.2% | 63.0% |
| Homeownership rate^1^ | 68.8% | 68.9% | 68.8% | 71.6% | 55.8% | 71.3% | 63.3% | 70.3% | 61.3% |
| Population with a disability^1^ | 18.1% | 21.9% | 17.3% | 18.5% | 14.6% | 25.9% | 17.6% | 13.5% | 16.4% |
| Without health insurance^1^ | 5.4% | 5.4% | 5.9% | 4.3% | 6.6% | 4.8% | 6.9% | 3.8% | 6.0% |

^1^Source: United States Census Bureau. (2024). Census Bureau Profiles. <https://data.census.gov/profile/>. Statistics reflect either 2020 Decennial Census (for population, race, ethnicity) or the 2022 American Community Survey (ACS) 5-Year Estimates (all other characteristics). Accessed on December 3, 2024.

^2^Source: Ingram, D. D., & Franco, S. J. (2014). 2013 NCHS Urban-Rural Classification Scheme for Counties. *Vital Health Statistics 2*(166), 1-73.

^3^The category of Other sums the US Census categories of American Indian and Alaskan Native, Native Hawaiian and Other Pacific Islander, and Some Other Race.
