# Supplementary material for: Organizational perspectives on the impacts of scaling up overdose education and naloxone distribution in Kentucky
Source: Addict Sci Clin Pract. 2025 Mar 14;20:27. doi: 10.1186/s13722-025-00553-2 (PMC11907800; doi:10.1186/s13722-025-00553-2)
Supplement: Supplementary file 2 — Supplementary Material 2 [file 13722_2025_553_MOESM2_ESM.docx]

**Organizational Perspectives on the Impacts of Scaling Up Overdose Education and Naloxone Distribution in Kentucky**

**Additional File 2**

Hannah K. Knudsen, Sandra Back-Haddix, Shaquita Andrews-Higgins, Michael Goetz, Olivia A. Davis, Douglas R. Oyler, Sharon L. Walsh, and Patricia R. Freeman

University of Kentucky

**Codebook**

This codebook was developed for the HEALing Communities Study for the purpose of coding qualitative interviews conducted with organizational partners that implemented Menu 1 (overdose education and naloxone distribution, OEND) and Menu 2 (medication for opioid use disorder, MOUD) interventions from the ORCCA during the Wave 1 study period (January 2020 – June 2022). Each “parent code” has “child codes” and “grandchild codes” to account for several specific facets of the study. However, only one round of these interviews was conducted during HCS, so only Wave 1 communities are represented in this sample. Interviews were conducted approximately 6-8 months after the Wave 1 active study implementation period ended in order to understand partners’ perspectives on implementation and early sustainability of the interventions. In Kentucky, interviews were conducted by Implementation Facilitators, most of whom were involved in helping organizational partners to implement EBPs from the two menus.

This codebook focuses on the OEND-Effectiveness code and presents additional child codes within the OEND-Effectiveness code to support sub-coding of impacts as reported by HCS-Kentucky (HCS-KY) partner organizations that implemented OEND. These child codes were developed deductively by an HCS-KY Co-investigator who initially read all passages of OEND Effectiveness that had been coded in the KY data as part of the cross-site process. After reading the passages, initial drafts of child code definitions and inclusion/exclusion criteria were developed. Then a small group of coders (who had been involved in conducting the Partner Sustainability interviews and/or initial cross-site coding) used the initial draft of OEND-Effectiveness child codes to code a set of passages to ensure consensus in application of the codes, to identify revisions to increase clarity of the codebook, and to determine whether additional child codes emerged from the data. This codebook development process was similar to the process described in McAlearney and colleagues [1].

In this codebook, each code and sub-code includes a definition and inclusion/exclusion criteria to assist in appropriately coding passages from the transcripts. A log of discrepancies and comments was included in a separate document as a means of tracking points of issue or discussion. This codebook was updated as coding progressed.

| **OEND Effectiveness** | Definition: Statements about the impacts/outcomes of any ORCCA opioid education and naloxone distribution (OEND) interventions on reducing overdose deaths and related outcomes.   - Possible outcomes include reducing deaths, reduction in overdoses, increased accessibility of Naloxone, and delivery of OEND.   Inclusion criteria:   - Include other proximal outcomes that are not the primary outcome of the intervention, i.e., stigma, norms, attitudes, increased accessibility and awareness of naloxone. - Include discussions of the impact of OEND strategies on the people served by the organization. - Include descriptions of ways that the OEND interventions impacted the organization itself, such as changing organizational culture, reducing stigma within the organization - Include discussions or mentions of non-fatal overdose rates. - Include statements in which an individual says “I don’t know” when asked about the impact or relative effectiveness of OEND interventions.   Exclusion criteria:   - No specific exclusions.   **NOTE:** No changes have been made to this code (i.e., this is the cross-site Partner Sustainability Interview Codebook version). Passages have already been coded to OEND Effectiveness, so the goal is to use the sub-codes below for finer-grained analysis. |
| --- | --- |
| **Lives Saved**  [Child code of OEND Effectiveness] | Definition: Statements about naloxone from the agency’s OEND program being used to save lives and reverse overdoses in the community.  Inclusion criteria:   - Include discussions of how the agency’s naloxone was used in ways that saved lives. - Include general statements about lives being saved in the community because of the agency’s OEND program (i.e., not a specific story but an overall summary of lives being saved because of partnering with HCS on OEND). - Include discussions of the agency’s naloxone being used during an overdose.   Exclusion criteria:   - No specific exclusions.   Opportunity for Double Coding   - There may be statements about a client who used their naloxone to save someone else’s life in their social network. This may be an opportunity to double code to Lives Saved and Social Networks of Clients/Patients |
| **Client Access and Repeated Requests**  [Child code of OEND Effectiveness] | Definition: Descriptions of the agency’s clients have greater access to naloxone because of the agency’s OEND program.  NOTE: “Clients/patients” throughout the codebook refers to individuals that the agency are likely to have multiple contacts/touchpoints with.  Inclusion criteria:   - Include descriptions focused on how the agency’s OEND program increased access to naloxone for agency’s clients/patients (e.g., “put it in our patient’s hands”). - Include stories of agency’s clients/patients asking for more units of naloxone after their initial receipt of OEND.   Exclusion criteria:   - Exclude statements about how the agency’s OEND program impacted other organizations or other locations that are part of their parent organization, and instead code to Organizational Networks. - Exclude statements about how the agency’s OEND program impacted naloxone access in the broader community, and instead code to Community Access.   Opportunity for Double Coding:   - There may be statements about how the agency’s OEND program impacted individuals connected to the agency’s patients/clients (e.g., family, friends) which resulted in additional requests for naloxone; in such cases, consider double coding to Client Access and Repeated Requests as well as Social Networks of Individuals. - There may be times that statements are interwoven between the organization’s clients and the community’s access; in such cases, consider double coding to Client Access and Repeated Requests as well as Community Access. |
| **Patient/Client Perceptions of Safety/Self-Efficacy**  [Child code of OEND Effectiveness] | Definition: Statements about how the agency’s OEND program impacted perceptions of readiness to respond to an overdose (e.g., safety, self-efficacy, and/or self-confidence) among the people that the agency serves (patients, clients).  Inclusion criteria:   - Include descriptions of how clients/patients felt more safe because they had received OEND. - Include descriptions of how clients/patients felt more prepared or more ready to respond to an overdose. - Include descriptions of how clients/patients felt more confident that they could effectively reverse an overdose using naloxone.   Exclusion criteria:   - Exclude statements about how the agency’s OEND program impacted family members’ feelings of safety, self-efficacy, or confidence and instead code to Social Networks of Individuals. - Exclude statements about how OEND improved relationships (e.g., trust, perceptions of care) between staff and patients/clients, and instead code to Clinical Relationships. |
| **Patient/Client Stigma Related to OEND**  [Child code of OEND Effectiveness] | Definition: Statements about how the agency’s OEND program impacted stigma from the perspective of the agency’s patients/clients, particularly emotional feelings of comfort, fear, etc.  Inclusion criteria:   - Include descriptions of how clients/patients became less fearful about requesting naloxone. - Include descriptions of how clients/patients became more willing to carry naloxone or receive OEND. - Include descriptions of how clients/patients can avoid stigma in the community because they can access OEND on-site at the agency. (Note: this passage should be also coded “Community Stigma Related to OEND”, e.g., "I've received so much stigma at the pharmacies so am glad I can now receive Narcan from this treatment center, where there is no stigma at all.")   Exclusion criteria:   - Exclude statements of how patients’ relationships to clinic staff improved and instead code to Clinical Relationships. |
| **Social Networks of Clients/Patients**  [Child code of OEND Effectiveness] | Definition: Statements about how the social networks of OEND recipients were impacted by the agency’s OEND program.  Inclusion criteria:   - Include descriptions of OEND recipients engaging in secondary distribution of naloxone (i.e., passing their naloxone to someone else in their social network such as family members, friends, coworkers; leaving naloxone units in locations where other people can access it). - Include descriptions of OEND recipients providing overdose education to other people in their social networks. - Include descriptions of agency staff providing OEND to the social networks of their clients/patients (i.e., to concerned family members who are not technically the agency’s clients/patients).   Exclusion criteria:   - Exclude statements about how the agency delivered OEND to the general public (i.e., non-clients/non-patients), and instead code to Community Access.   Opportunity for Double Coding   - There may be statements about a client who used their naloxone to save someone else’s life in their social network. This may be an opportunity to double code to Lives Saved and Social Networks of Clients/Patients. |
| **Clinical Relationships**  [Child code of OEND Effectiveness] | Definition: Statements about how the agency’s OEND program impacted relationships between agency staff and the people that they serve.  Inclusion criteria:   - Include descriptions of how implementing OEND impacted the extent to which patients/clients trusted the agency and/or its staff. - Include discussions of how implementing OEND resulted in patients/clients perceiving that agency staff cared about them and their well-being.   Exclusion criteria:   - Exclude statements about how clients became less fearful about requesting naloxone from agency staff and instead code to Patient/Client Stigma. |
| **Staff Perceptions of Safety/Self-Efficacy**  [Child code of OEND Effectiveness] | Definition: Statements about how the agency’s OEND program impacted staff perceptions of readiness to respond to an overdose (e.g. safety, self-efficacy, and/or self-confidence) among agency staff (which would include Voices of Hope and/or Bluegrass Care Navigator staff who were placed at the agency).  Inclusion criteria:   - Include descriptions of how staff felt more safe because they had received OEND. - Include descriptions of how staff felt more prepared or more ready to respond to an overdose. - Include descriptions of how staff felt more confident that they could effectively reverse an overdose using naloxone.   Exclusion criteria:   - Exclude statements about how OEND improved relationships (e.g., trust, perceptions of care) between staff and patients/clients, and instead code to Clinical Relationships. |
| **Staff Stigma Related to OEND**  [Child code of OEND Effectiveness] | Definition: Statements about how the agency’s OEND program impacted stigma from the perspective of the agency’s staff (e.g., in general or among certain types of staff such as MOUD providers, harm reduction staff, counselors, etc.), particularly negative emotional feelings/attitudes about OEND.  Inclusion criteria:   - Include descriptions of how staff attitudes toward OEND changed through the experience of the agency implementing OEND. - Include descriptions of how staff became more open to OEND and/or harm reduction because the agency implemented OEND. - Include descriptions of cultural change within the agency because of implementing OEND. - Include descriptions of staff becoming more aware of the needs of people with OUD because of implementing OEND.   Exclusion criteria:   - Exclude statements describing how the agency’s OEND program impacted the attitudes of staff at other agencies, and instead code to Organizational Networks. |
| **Organizational Networks**  [Child code of OEND Effectiveness] | Definition: Statements about how the agency’s OEND program or the agency’s staff impacted other organizations in the community.  Inclusion criteria:   - Include statements about how the agency trained other organizations (or staff from other sites of their larger organization) about OEND. - Include statements about how the agency shared information about how to access a supply of naloxone (e.g., from KORE) with other organizations and/or other sites of their larger organization. - Include descriptions of how the agency’s implementation of OEND impacted partnerships with other agencies. - Include descriptions of how the agency’s implementation of OEND impacted the attitudes of staff who work in other agencies.   Exclusion criteria:   - Exclude descriptions of how the agency’s OEND program increased access to members of the broader community, and instead code to Community Access. - Exclude descriptions of how the agency’s implementation of OEND impacted general community attitudes (i.e., without reference to specific other agencies), and instead code to Community Stigma. |
| **Community Access**  [Child code of OEND Effectiveness] | Definition: Statements about how the agency’s implementation of OEND impacted access to naloxone in the broader community.  Inclusion criteria:   - Include descriptions of the agency doing direct delivery of OEND at events/locations in the community. - Include discussions of general changes in OEND access in the community because of the agency’s OEND program.   Exclusion criteria:   - Exclude descriptions of how the agency’s OEND increased access to naloxone specific to their own patients/clients, and code to Client Access and Repeated Requests.   Potential for Double Coding:   - There may be times that statements are interwoven between the organization’s clients and the community’s access; in such cases, consider double coding to Client Access and Repeated Requests as well as Community Access. |
| **Community Stigma Related to OEND**  [Child code of OEND Effectiveness] | Definition: Statements about how the agency’s OEND program has impacted stigma in the broader community.  Inclusion criteria:   - Include descriptions of how clients/patients can avoid stigma in the community because they can access OEND on-site at the agency. (Note: this passage should be also coded “Patient/Client Stigma Related to OEND”, e.g., "I've received so much stigma at the pharmacies so am glad I can now receive Narcan from this treatment center, where there is no stigma at all.") - Include descriptions of how carrying naloxone has become less stigmatized in the broader community. - Include statements about how more ordinary citizens or members of the general public are willing to carry naloxone. - Include general statements about changing public attitudes toward OEND because of the agency’s OEND program.   Exclusion criteria:   - Exclude discussions of how organizational partnerships around OEND have impacted stigma/attitudes of the staff of other organizations, and instead code to Organizational Networks. |
| **Uncertainty of OEND Impacts**  [Child code of OEND Effectiveness] | Definition: Descriptions of uncertainty about the impacts of the agency’s OEND program.  Inclusion criteria:   - Include statements indicating that the interviewee is unsure of the OEND program’s impacts on individuals, organizations, and/or the community. - Include statements about lack of certainty about whether the agency’s OEND program has saved lives.   Exclusion criteria:   - No specific exclusions. |

**References**

1. McAlearney AS, Walker D, Shiu-Yee K, Crable E, Auritt V, Barkowski L, Batty E, Dasgupta A, Goddard-Eckrich D, Knudsen HK, et al. Embedding big qual and team science into qualitative research: Lessons from a large-scale, cross-site research study. International Journal of Qualitative Methods. 2023;22:1-9.
