# Supplementary material for: Organizational perspectives on the impacts of scaling up overdose education and naloxone distribution in Kentucky
Source: Addict Sci Clin Pract. 2025 Mar 14;20:27. doi: 10.1186/s13722-025-00553-2 (PMC11907800; doi:10.1186/s13722-025-00553-2)
Supplement: Supplementary file 3 — Supplementary Material 3 [file 13722_2025_553_MOESM3_ESM.docx]

**Organizational Perspectives on the Impacts of Scaling Up Overdose Education and Naloxone Distribution in Kentucky**

**Additional File 3**

Hannah K. Knudsen, Sandra Back-Haddix, Shaquita Andrews-Higgins, Michael Goetz, Olivia A. Davis, Douglas R. Oyler, Sharon L. Walsh, and Patricia R. Freeman

University of Kentucky

**Consolidated Criteria for Reporting Qualitative Studies (COREQ): 32-item checklist**

**Domain 1: Research Team and Reflexivity**

***Personal Characteristics***

**1. Which author/s conducted the interview or focus group?**

Authors who conducted interviews

Sandra Back-Haddix

Shaquita Andrews-Higgins

Michael Goetz

Non-Authors who conducted interviews (but not analysis)

Kathy Adams

Jeannie Hartman

Rachel Hoover

Latasha Jones

Hallie Mattingly

Ryan Morris

Melissa Reedy-Johnson

Please see the manuscript’s Acknowledgements and Contributions sections for more information.

**2. What were the researcher’s credentials? E.g. PhD, MD.**

The authors’ credentials are below:

Hannah K. Knudsen, PhD

Sandra Back-Haddix, MPH

Shaquita Andrews-Higgins, LPCA

Michael Goetz, PhD

Olivia A. Davis, BS

Douglas R. Oyler, PharmD

Sharon L. Walsh, PhD

Patricia R. Freeman, RPh, PhD, FAPhA, FNAP

Of non-authors who conducted interviews but not analysis, all were master’s level staff who worked on the project.

**3. What was their occupation at the time of the study?**

Faculty at the University of Kentucky: Hannah K. Knudsen, Patricia R. Freeman, Douglas R. Oyler, Sharon L. Walsh

Implementation Facilitator/Researcher at the University of Kentucky: Shaquita Andrews-Higgins, Sandra Back-Haddix, Michael Goetz

Medical Student at the University of Kentucky College of Medicine: Olivia Davis

Of non-authors who conducted interviews but not analysis, all were Implementation Facilitators.

**4. Was the researcher male or female?**

Data collection, coding, analysis and manuscript preparation were conducted collaboratively by diverse teams comprising both women and men.

**5. What experience or training did the researcher have?**

Interviewers were trained by faculty from the four state research teams (KY, MA, NY, OH) in a cross-site interview guide that included questions and follow-up probes. Faculty trained interviewers on how to use the interview guide, including techniques to elicit useful information pertinent to the study. A cross-site consensus-based process was used for initial coding, as described in McAlearney et al. and described in the manuscript. Interviewers and a bachelor’s-level administrative assistant engaged in the initial coding after being trained on the cross-site codebook; a similar process was used for three previous rounds of interviews before the current set of interviews were coded. For the additional inductive coding, development of the codebook and training is described in the Methods section of the paper.

**6. Was a relationship established prior to study commencement?**

Relationships with participants were not established prior to the start of the HEALing Communities Study (HCS). However, some interviewers had worked with interviewees on the implementation of evidence-based practices as part of HCS, which occurred prior to the conduct of the interviews described in this manuscript. This is noted in the Limitations section of the manuscript.

**7. What did the participants know about the researcher? e.g. personal goals, reasons for doing the Research.**

Participants in this research were aware that the researchers aimed to address opioid overdoses; however, personal goals or motivations were not a focus of discussion.

**8. What characteristics were reported about the interviewer/facilitator? e.g. Bias, assumptions, reasons and interests in the research topic.**

None.

**Domain 2: Study Design**

***Theoretical framework***

**9. What methodological orientation was stated to underpin the study? e.g. grounded theory, discourse analysis, ethnography, phenomenology, content analysis.**

Methodological orientation (i.e., thematic analysis) is described in the Methods section.

**10. How were participants selected? e.g. purposive, convenience, consecutive, snowball.**

The purposive interview participant selection process is detailed in the Methods section.

**11. How were participants approached? e.g. face-to-face, telephone, mail, email.**

Participants were contacted by email or phone for recruitment. This process is detailed in the methods section.

**12. How many participants were in the study?**

This manuscript provides documentation of interview participants (n=70), along with associated demographic characteristics (see Table 1).

**13. How many people refused to participate or dropped out? Reasons?**

The number of people who refused to participate after interview request is detailed in the paper (n=14 active refusals; n=39 who did not respond to repeated invitations). This was a cross-sectional study so there was no dropout that occurred.

***Setting***

**14. Where was the data collected? e.g. home, clinic, workplace.**

Interviews were conducted via Zoom video conference calls by interviewers in their remote offices. Participants could choose where to participate in the Zoom calls; participants were largely interviewed at their employing organization.

**15. Was anyone else present besides the participants and researchers?**

There may have been additional individuals near the participant during the interview, but given the reliance on Zoom, it was not possible for the interviewer to determine if this was the case. This information was not recorded.

**16. What are the important characteristics of the sample? e.g. demographic data, date.**

Demographics are described in the manuscript in Table 1.

***Data collection***

**17. Were questions, prompts, guides provided by the authors? Was it pilot tested?**

Interviewers were provided with an interview guide with prompts. The guide was not formally pilot tested, but interviewers did practice prior to conducting interviews.

**18. Were repeat interviews carried out? If yes, how many?**

Repeat interviews were not carried out.

**19. Did the research use audio or visual recording to collect the data?**

Yes. All interviews were audio- and video-recorded via the Zoom interface, but only the audio recording was transcribed, coded, and analyzed.

**20. Were field notes made during and/or after the interview or focus group?**

Outside of demographic information collected during and/or after recorded interview, field notes were the choice of the researcher and were not included in any data analyzed.

**21. What was the duration of the interviews or focus group?**

As mentioned in the manuscript, the interviews varied in length but averaged 60 minutes long.

**22. Was data saturation discussed?**

No. As noted in the manuscript, data saturation was not feasible for the scale of this data collection across the four states. Purposive sampling was used to identify a group of potential interviewees, and all were invited to participate. All data were coded and used in the analysis.

**23. Were transcripts returned to participants for comment and/or correction?**

As the coding, analysis, and manuscript preparation occurred after the intervention concluded, we did not re-contact interviewees to provide feedback on the transcripts or the manuscript itself.

**Domain 3: Analysis and Findings**

***Data analysis***

**24. How many data coders coded the data?**

The number of coders and the process is detailed in the manuscript.

**25. Did authors provide a description of the coding tree?**

We did not provide a coding tree, *per se*, but did provide the study’s codebook, which indicates codes, child codes, and grandchild codes associated with themes within the research (see Additional File 2).

**26. Were themes identified in advance or derived from the data?**

Themes for this study were derived from the data (i.e., inductively).

**27. What software, if applicable, was used to manage the data?**

NVivo 12 was used to manage the qualitative transcripts. REDCap 14.4.1 was used to enter the demographic data and track participation; these data were exported to Stata 18 to calculate descriptive statistics.

**28. Did participants provide feedback on the findings?**

No.

***Reporting***

**29. Were participant quotations presented to illustrate the themes / findings? Was each**

**quotation identified? e.g. participant number**

Yes.

**30. Was there consistency between the data presented and the findings?**

Yes.

**31. Were major themes clearly presented in the findings?**

Yes.

**32. Is there a description of diverse cases or discussion of minor themes?**

A comprehensive discussion of minor themes is not included although these themes are referenced.
