# Supplementary material for: Organizational perspectives on the impacts of scaling up overdose education and naloxone distribution in Kentucky
Source: Addict Sci Clin Pract. 2025 Mar 14;20:27. doi: 10.1186/s13722-025-00553-2 (PMC11907800; doi:10.1186/s13722-025-00553-2)
Supplement: Supplementary file 4 — Supplementary Material 4 [file 13722_2025_553_MOESM4_ESM.docx]

**Organizational Perspectives on the Impacts of Scaling Up Overdose Education and Naloxone Distribution in Kentucky**

**Additional File 4**

Hannah K. Knudsen, Sandra Back-Haddix, Shaquita Andrews-Higgins, Michael Goetz, Olivia A. Davis, Douglas R. Oyler, Sharon L. Walsh, and Patricia R. Freeman

University of Kentucky

**Additional Passage Supporting the Themes**

| **Theme** | **Additional Passages** |
| --- | --- |
| Lives Saved and Increased Access | *“I think that, on my end, I felt like that it [implementing OEND] did two things when people initially came. And that was to communicate very clearly the value that we place on harm reduction and also the degree to which we valued their safety. Taking the time, prioritizing that as something that is just part of our expectations in terms of their trajectory of care. And so, I think that making that, demonstrating that was valuable to us, helped it be a priority to some patients that maybe it wouldn't have necessarily been. The other thing is that it resulted in multiple saved lives. We had multiple people that came back and said, "Thank you so much. I had to use it twice," or whatever.”*  *(01072807)*  *“So having that Spanish interpretation or Swahili was wonderful as well, that we didn't have to do it through a CyraCom [medical translation service] or anything like that. The patients really appreciated that we were able to provide their language in real-time.”*  *(01074356)*  *“And like I said, we have truly had people come back in and say, "I am so glad that I took that Narcan because I saved somebody at work. I saved my friend," that type of thing. So it is wonderful, and I feel very, very blessed that we have had that to hand out personally here.”*  *(02030215)*  *“We'll continue to reach out to maybe said family members or whatever. Yeah, that's going to be something that we continue. As long as they will give it to us, we'll probably continue to push it out. Especially with the rise of fentanyl deaths that we've seen and overdoses lately here in this area. I would hope that each participant would have at least 1 to maybe 2 boxes of Narcan due to how strong the fentanyl is.”*  *(01082056)*  *“Very successful. I think we're continuing to use it now, even with KORE [Kentucky Opioid Response Effort]. It's something that our patients utilize, use on a regular basis. They always know that they can come here and get some. So it's very - I think that opened doors for discussion and just people being more comfortable asking for that resource, knowing it's going to happen out there in the real world. So I think just making that comfortable place for them.”*  *(01072808)*  *“We had somebody from the community come in and knock on our door. She was like, "I've got somebody in my car who's overdosing right now." I kept my kit down in the med staff area with their medical supplies, and our med staff went out there with that kit and thankfully saved that gentleman's life. I don't know where they are now. I would encourage them to come in and see us in the future, but I'm not sure what ended up happening with them, but it was a huge benefit for us to have it and for the patients that we've talked to that were able to get it from us, and have been able to keep coming back to us to get more, if they needed it. Of course we have them fill out another demographic sheet.”*  *(01072805)* |
| Patient/Client Stigma and Perceptions of Safety/Self-Efficacy | *“Interviewee: The people that are stable, you know, I think they tend to decline it more than not. “And even if, just for when they complete the program, if they were to relapse, they're at much greater risk for overdose. So, we try to educate them on that and still offer it that time, if that makes sense.*  *Interviewer: Do you think there was some stigma with the patient on carrying it? Just that fear that it would look like they were going to relapse?*  *Interviewee: For some. I would say some of them were like, "Oh, I don't need that. I'm okay," but then we also had other patients who wanted to carry it just in case they ran across somebody on the bus, or they just wanted to have it while they were out in the community, so kind of depended on the person.”*  *(01132432)*  *“We want everybody to be safe and feel like they have the tools that they need for wherever they are on their recovery journey. And this is just one of those tools. Same as therapy or group or whatever it may, peer support, whatever it may be, case management. I guess it's just another tool that we can offer you in that journey.”*  *(01152446)*  *“I think on a bigger scale, because you have to believe that even though we may serve 125 people a month, there's other people who aren't visiting me that are actively using, and whether they share supplies within their group or they split the supplies that they're given just because for whatever reason, that person is still unwilling to come up here and receive services, that's fine. I wholeheartedly believe that that community of people who use drugs is so interwoven that not only did they probably already know how to use Narcan, but I have to believe that some of that overdose education and the awareness made about rescue breaths two to three minutes, and the actual way that it's supposed to be done, I have no doubt that they shared that within their community of other people who use drugs within the community.”*  *(01031179)* |
| Staff Perceptions of Safety/Self- Efficacy | *“I feel more comfortable having it [naloxone] here. I'm sure that the rest of the staff feels the same way, because before we weren't even allowed to administer it, even if we had it on our own, like in our car. We weren't allowed to administer it because we weren't trained. Yeah. I feel a lot more... like just now, I'm like, "Okay, where's my Narcan?" I feel more comfortable. I don't feel as worried about it.”*  *(01092415)*  *“Well, it opened a door for me. I wasn't really aware of Narcan or how it worked or anything like that, even though I've been in addiction, but I was never around it or ever heard of it. And so, it brought a lot of education to me and that allowed me to just share it with other people. So, I think it's helped a lot.”*  *(01082401)* |
| Clinical Relationships | *“They always know that they can come here and get some. So, it's very - I think that opened doors for discussion and just people being more comfortable asking for that resource, knowing it's going to happen out there in the real world. So, I think just making that comfortable place for them.”*  *(01072808)*  *“And then with the patients, them feeling comfortable enough to come and say like, "Hey, I think I need this, or I'm still in interaction or communication with people that I feel like I need to have this to be able to save their life." It was just building a respect between us and the patients of, we weren't judging them. We were truly there to let them know that we were working to save their life and part of their recovery journey, and that was simply it.”*  *(01152446)* |
| Staff Stigma Related to OEND | *“And that was something that’s honestly really important to us at [primary care clinic]: we take care of these really vulnerable patients, and we want the staff to-- even if you have personal biases against people that are using substances--know that we are here to serve everyone in our community. And that means that that’s likely going to be someone you’re going to serve. So, it was a really cool shift to see that in some of our staff.”*  *(01151015)*  *“Behavioral health and medical is so different. And I don't want to say that that Narcan or naloxone was a taboo thing, but it was just something like the doctors were really accustomed to that opioid prescription and that kind of being it, "We're going to help you control your pain. We're here for that," but taking it the next step further and just realizing that there is a potential for an accident, not only for the patient but for people in the home, I think has been really rewarding for the behavioral health side. And it's been really helpful for the medical side just to see the full picture. It doesn't just end writing that script. There's way more to it. So I think that's been a big benefit all the way around.”*  *(01070983)* |
| Impacts on Organizational Networks | *“So, once we explained it to them, the need showing the numbers of overdoses in this area, they got on board with doing a training of all court staff that dealt with specialty courts. So, we did that first. We reached out and spoke to, of course, the people that are going to be the first to respond, usually our fire departments, our police officers, and the jail staff.”*  *(01082056)*  *“Interviewer: Well, I called it from the beginning that you’re going to become a model for other health departments, especially, to launch this type of program. I think you’re going to find that increasingly is the case. You’re going to get more and more phone calls.*  *Interviewee: Yeah. No, I think it’s definitely. We’ve seen that. That’s why I told the staff, I was like, “Let’s break this down. I just want to show where our footprint is and see— “. Because initially, we were getting these requests, but yeah. When staff were like, “Oh, we’re getting Texas,” or different places, they’re like, “This is crazy.”*  *(01131207)* |
| Community Access | *“One guy was coming in to pick up prescriptions for his daughter. And he said that he had experienced an overdose while at work. Someone on his line at the factory he worked in had overdosed. So, he would like to get some just in case. So, the majority of the individuals that were actually getting it through this project were not patients of ours.”*  *(01151015)*  *“People were actually, they kind of knew that we had the Narcan or that I had the Narcan and that I could provide the training and they would reach out to me, whether it be on social media or whatever, and ask "Where can I get this?" And so I think that it really impacted our whole community with their access and education to Narcan.”*  *(01152791)* |
| Reductions in Community Stigma | *“Interviewee 1: Naloxone and saving lives that was the biggest impact. But education and prevention, too.*  *Interviewee 2: Well, and not just getting Narcan. Not just getting the Narcan in their hands, but the education that people got with that. Because they began to accept it in that process. It became much more acceptable. So, that made a big difference.”*  *(01022769)*  *“I don't know if I can relate it necessarily back to the HEALing Communities Study, but I think that one of the things we mentioned earlier was there does seem to be less of, just across the board, less stigma associated with carrying Narcan and to the degree that the HEAL Study participated in making that available. And normalizing it some, I think that that would be an impact on the community.”*  *(01072807)*  *“It also gave people a sense of power. The situation is you're really powerless in this situation. Some people don't care, but some people feel this urge. Like I have no role in helping my community… but giving them Narcan gives them a little comfort knowing I have a role now. If it's this small, this is my role.”*  *(01022769)* |
